# Overexpression of Delayed Rectifier K^+^ Channels Promotes *In situ* Proliferation of Leukocytes in Rat Kidneys with Advanced Chronic Renal Failure

**DOI:** 10.1155/2012/581581

**Published:** 2012-05-31

**Authors:** Itsuro Kazama, Yoshio Maruyama, Yasuhiro Endo, Hiroaki Toyama, Yutaka Ejima, Mitsunobu Matsubara, Shin Kurosawa

**Affiliations:** ^1^Department of Physiology I, Tohoku University Graduate School of Medicine, Seiryo-cho, Aoba-ku, Sendai, Miyagi 980-8575, Japan; ^2^Department of Anesthesiology, Tohoku University Hospital, Seiryo-cho, Aoba-ku, Sendai, Miyagi 980-8574, Japan; ^3^Division of Molecular Medicine, Center for Translational and Advanced Animal Research, Tohoku University Graduate School of Medicine, Seiryo-cho, Aoba-ku, Sendai, Miyagi 980-8575, Japan

## Abstract

Leukocytes, such as lymphocytes and macrophages, predominantly express delayed rectifier K^+^ channels (Kv1.3), and the channels play crucial roles in the activation and proliferation of the cells. Since lymphocytes are activated in patients with end-stage renal disease (ESRD), the channels expressed in those cells would contribute to the progression of renal fibrosis in advanced-stage chronic renal failure (CRF). In the present study, using a rat model with advanced CRF that underwent 5/6 nephrectomy followed by a 14-week recovery period, we examined the histopathological features of the kidneys and the leukocyte expression of Kv1.3-channels and cell cycle markers. Age-matched sham-operated rats were used as controls. In the cortical interstitium of advanced CRF rat kidneys, leukocytes proliferated *in situ* and overexpressed Kv1.3 channel protein in their cytoplasm. Treatment with margatoxin, a selective Kv1.3-channel inhibitor, significantly suppressed the number of leukocytes and the progression of renal fibrosis with a significant decrease in the cortical cell cycle marker expression. This study demonstrated for the first time that the number of leukocytes was dramatically increased in rat kidneys with advanced CRF. The overexpression of Kv1.3 channels in the leukocytes was thought to contribute to the progression of renal fibrosis by stimulating cell cycling and promoting cellular proliferation.

## 1. Introduction

Chronic renal failure (CRF) progresses relentlessly to end-stage renal disease (ESRD). In patients with advanced CRF, the disease tends to progress faster than in those with mild to moderate CRF [1, 2]. Although several factors, such as advanced age, hypertension, dehydration, and the use of drugs, are known to affect the rate of progression [3–5], it can spontaneously accelerate despite the absence of such aggravating factors [1]. These findings indicate the involvement of an additional mechanism that promotes the progression of CRF in the advanced-stage. The histopathology of kidneys in CRF is characterized by tubulointerstitial fibrosis in both humans [6, 7] and experimental animal models [8–10]. In the development of renal fibrosis, previous studies have revealed the initial involvement of inflammatory leukocytes, such as lymphocytes, macrophages, and mast cells [11, 12]. However, we know little about their later involvement in the progression of renal fibrosis in the advanced-stage, partly because of the high mortality rate of the experimental animals [11]. Lymphocytes and macrophages predominantly express delayed rectifier K^+^-channels (Kv1.3) in their plasma membranes, and the channels play crucial roles in the activation and proliferation of the cells [13–16]. Since lymphocytes are activated [17] and serum cytokine levels are much elevated in patients with ESRD [18, 19], the channels expressed in the leukocytes would contribute to the progression of renal fibrosis in advanced CRF. To elucidate this, using a rat model with advanced CRF, we examined the histopathological features of the kidneys and the involvement of Kv1.3 channel and cell cycle marker expression in the pathogenesis. Here, we show for the first time that the number of leukocytes dramatically increased in rat kidneys with advanced CRF. We also show that the overexpression of Kv1.3 channels in the leukocytes contributed to the progression of renal fibrosis by stimulating cell cycling and promoting cellular proliferation.

## 2. Materials and Methods

### 2.1. Animal Preparation

Rats with 5/6 nephrectomy with recovery periods as long as 14 weeks were used as the model of advanced CRF in the present study. Subtotal nephrectomy was performed in male Sprague-Dawley rats weighing 150–180 g (Japan SLC Inc., Shizuoka, Japan) as described in our previous study [20]. Briefly, the upper 1/3 and lower 1/3 of the right kidney were ligated to induce infarction. One week later, the left kidney was removed. During the subsequent 14 weeks, rats had free access to standard rat chow and water ad libitum and were maintained in a humidity- and temperature-controlled room on a 12-hour light-dark cycle. Age-matched sham operated rats were used as controls. For the treatment with Kv1.3 channel inhibitor, margatoxin (Peptide Institute, Osaka, Japan) was dissolved in distilled water to prepare a concentration of 100 nM. Twelve weeks after subtotal nephrectomy, CRF rats were intraperitoneally injected with 100 nM/mL margatoxin daily for two weeks. At the end of the 14-week recovery period, the rats were deeply anaesthetized with isoflurane and then killed by cervical dislocation. Trunk blood was withdrawn for the measurements of serum creatinine, urea nitrogen, and potassium levels. Kidneys were removed for histological examination and RNA extraction. All experimental protocols described in the present study were approved by the Ethics Review Committee for Animal Experimentation of Tohoku University.

### 2.2. Immunohistochemistry

Three-micrometer paraffin sections of 4% paraformaldehyde-fixed kidneys were placed in citrate-buffered solution (pH 6.0) and then boiled for 30 min for antigen retrieval. Endogenous peroxidase was blocked with 3% hydrogen peroxide, and nonspecific binding was blocked with 10% BSA. Primary antibodies were as follows: rabbit anti-Ki-67 (1 : 100; Lab Vision Co., Fremont, CA, USA), anti-Kv1.3 (1 : 100; Alomone Labs Ltd., Jerusalem, Israel), and mouse anticollagen type III (1 : 100; Abnova, Taipei City, Taiwan). Diaminobenzidine substrate (Sigma Chemical Co., St. Louis, MO, USA) was used for the color reaction. For Kv1.3 and collagen III staining, sections were counterstained with hematoxylin. Secondary antibody alone was consistently negative on all sections.

### 2.3. Real-Time RT-PCR

Total RNAs from freshly isolated renal cortex were extracted using the RNeasy mini kit (Qiagen, Hilden, Germany). First-stand cDNA was synthesized from 2 *μ*g of total RNA of each tissue in 20 *μ*L of reaction mixture using the SuperScript VILO first-strand synthesis kit (Invitrogen, Carlsbad, CA, USA). The quantitative RT-PCR was carried out using the Applied Biosystems 7500 Real-Time PCR System (Life Technologies Inc., Gaithersburg, MD, USA) with SYBR Premix Ex Taq II (Takara Bio, Kyoto, Japan). The quantity of RNA samples was normalized by the expression level of GAPDH. The sequences of the primers used are listed in Table 1.

### 2.4. Other Measurements and Statistical Analyses

Serum creatinine, urea nitrogen, and potassium levels were measured using a chemical autoanalyzer (DRI-CHEM 3500V; Fuji, Tokyo, Japan). Data were analyzed using Microsoft Excel (Microsoft Co., Redmond, WA, USA) and reported as means ± SEM. Statistical significance was assessed by a two-way ANOVA followed by Dunnett's or Student's *t*-test. A value of *P* < 0.05 was considered significant.

## 3. Results

### 3.1. In Situ Proliferation of Inflammatory Leukocytes in Rat Kidneys with Advanced CRF

**Figure 1:**
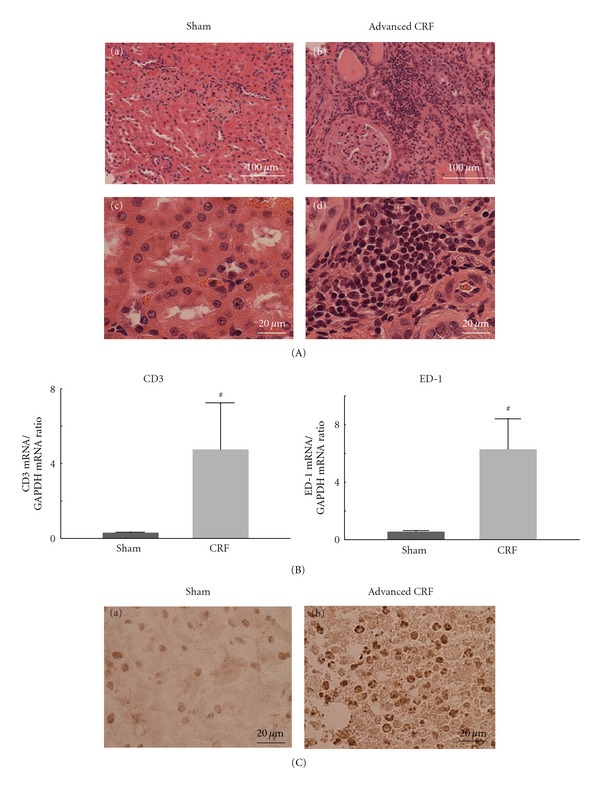
Histological features of sham operated (sham) and advanced CRF rat kidneys. (A) Hematoxylin and eosin staining (H&E) in sham operated (sham) and advanced CRF rat kidneys. (a) and (b) Low-power views of cortex. Magnification, ×20. (c) and (d) High-power views of cortical interstitium. Magnification, ×60. (B) The mRNA abundance of CD3 (left) and ED-1 (right) in the renal cortex of sham operated and advanced CRF (CRF) rat kidneys. ^#^ *P* < 0.05 versus sham operated rats. Values are means ± SEM (*n* = 6). Differences were analyzed by ANOVA followed by Dunnett's or Student's *t*-test. (C) Immunohistochemistry using antibody for Ki-67 (brown) in sham operated and advanced CRF rat kidneys. (a) and (b) High-power views of cortical interstitium. Magnification, ×60.

The marked elevation of serum creatinine (4.45 ± 1.16 versus sham operated 0.33 ± 0.03 mg/dL, *n* = 6, *P* < 0.05) and urea nitrogen (130 ± 8.95 versus sham operated 15.5 ± 1.39 mg/dL, *n* = 6, *P* < 0.05) levels in nephrectomized rats indicated advanced CRF with severe uremia [11]. The rats presented hyperkalemia (6.9 ± 0.5 versus sham operated 4.3 ± 0.05 mEq/L, *n* = 6, *P* < 0.05) as a result of deteriorated renal function. Sections of kidneys from sham operated rats showed normal tubulointerstitium of the cortex (Figures 1A(a) and 1A(c)). In CRF rat kidneys, as previously demonstrated [8, 10, 12], diffuse fibrosis was noted in the medullary and papillary interstitium (data not shown). In the cortical interstitium, in addition to fibrosis, a substantial number of small, round cells were noted among spindle-shaped fibroblasts (Figures 1A(b) and 1A(d)). Since the mRNA expression of CD3 and ED-1, surface markers for T-lymphocytes and macrophages, was markedly elevated in the cortex isolated from CRF rat kidneys (Figure 1(B)), those round cells were considered to be inflammatory leukocytes, such as T-lymphocytes and macrophages.

In sham operated rat kidneys, immunohistochemistry for Ki-67, a marker of cellular proliferation, was only weakly positive in some proximal tubular cells (Figure 1C(a)). In CRF rat kidneys, however, the immunohistochemistry demonstrated a number of positively stained small, round cells within the cortical interstitium (Figure 1C(b)), indicating that inflammatory leukocytes were proliferating in the kidneys of advanced CRF.

### 3.2. Overexpression of Kv1.3-Channels in the Leukocytes and Expression of Cell Cycle Markers

**Figure 2:**
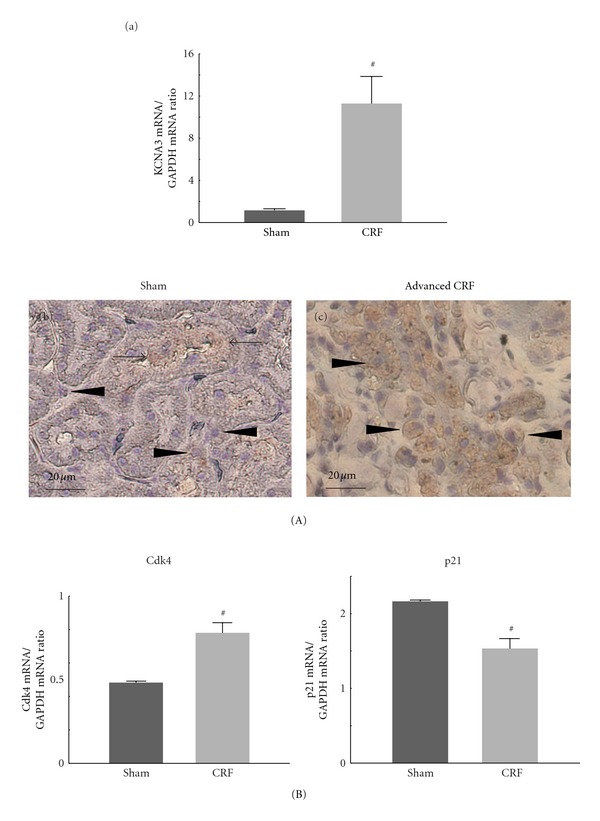
Kv1.3 and cell cycle marker expression in sham operated (sham) and advanced CRF rat kidneys. (A) Kv1.3 expression. (a) KCNA3 mRNA abundance in the renal cortex of sham operated (sham) and advanced CRF (CRF) rat kidneys. (b) and (c) Immunohistochemistry using antibody for Kv1.3 (brown) in sham operated and advanced CRF rat kidneys. High-power views of cortical interstitium. Magnification, ×60. (B) Cell cycle marker expression. The mRNA abundance of cyclin-dependent kinase 4 (Cdk4) (left) and p21 (right) in the renal cortex of sham operated and advanced CRF rat kidneys. ^#^ *P* < 0.05 versus sham operated rats. Values are means ± SEM (*n* = 6). Differences were analyzed by ANOVA followed by Dunnett's or Student's *t*-test.

Since lymphocytes and macrophages express Kv1.3 channels in their plasma membranes [13, 14], and since the channels trigger the calcium influx necessary for cellular proliferation [15, 16], we examined the expression of the channel in leukocytes of the rat kidneys (Figure 2(A)). The mRNA expression of *KCNA3* gene, which encodes Kv1.3, was markedly elevated in the cortex isolated from CRF rat kidneys (Figure 2A(a)). In sham operated rats, as previously demonstrated [21], immunohistochemistry for Kv1.3 showed a weak staining in the cytoplasm of some proximal tubular cells (Figure 2A(b), arrows), but not in small round cells in the cortical interstitium (arrow heads). In CRF rats, however, the cytoplasmic expression of Kv1.3 was increased specifically in small round cells proliferating in the cortical interstitium (Figure 2A(c), arrow heads), indicating the overexpression of the channels in the leukocytes.

Since overexpression of Kv1.3 channels is known to affect the cell cycle progression in various types of cancer cells [22, 23], we then examined the expression of cell cycle markers, such as cyclin-dependent kinase 4 (Cdk4) and its inhibitor, p21 (Figure 2(B)). In CRF rat kidneys, the cortical expression of Cdk4 mRNA was significantly increased, but that of p21 mRNA was significantly decreased compared to sham operated rat kidneys.

### 3.3. Effects of a Selective Kv1.3-Channel Inhibitor on Renal Fibrosis and Cell Cycle Marker Expression

**Figure 3:**
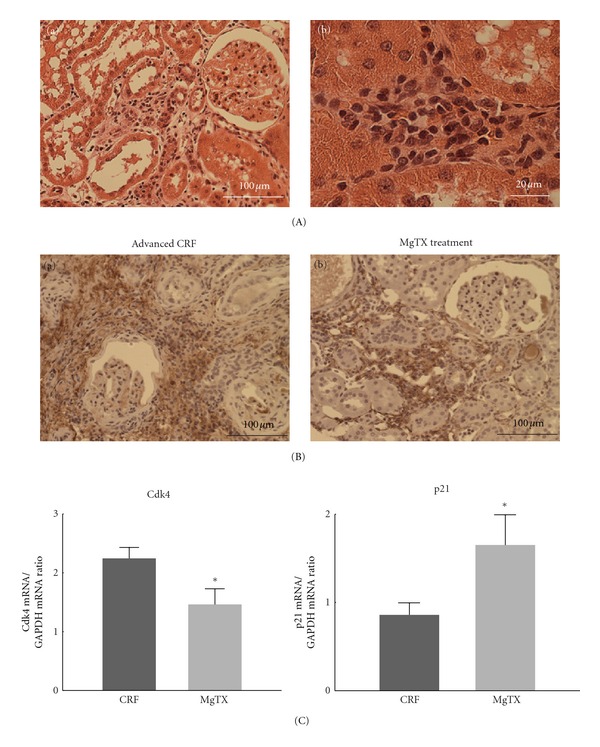
Collagen III and cell cycle marker expression in advanced CRF rat kidneys after margatoxin (MgTX) treatment. (A) Hematoxylin and eosin staining (H&E) in advanced CRF rat kidneys after margatoxin (MgTX) treatment. (a) A low-power view of cortex. Magnification, ×20. (b) A high-power view of cortical interstitium. Magnification, ×60. (B) Immunohistochemistry using antibody for collagen III (brown) in advanced CRF rat kidneys with and without MgTX treatment. (a) and (b) Low-power views of cortex. Magnification, ×20. (C) Cell cycle marker expression. The mRNA abundance of Cdk4 (left) and p21 (right) in the renal cortex of advanced CRF rat kidneys with and without MgTX treatment. *P < 0.05 versus advanced CRF rats without treatment. Values are means ± SEM (*n* = 6). Differences were analyzed by ANOVA followed by Dunnett's or Student's *t*-test.

To obtain the direct evidence that the overexpression of Kv1.3 channels actually contributes to the proliferation of leukocytes and to the progression of renal fibrosis, we finally examined CRF rat kidneys after treating with margatoxin, a selective Kv1.3 channel inhibitor [22, 24]. In CRF rat kidneys with margatoxin treatment, proximal tubules were partially preserved, although they were atrophic (Figure 3A(a)). Compared to CRF rat kidneys without treatment (Figures 1A(b) and 1A(d)), the size of the cortical interstitium was smaller (Figure 3A(a)), and the number of infiltrating leukocytes was much less (Figure 3A(b)). Immunohistochemistry for collagen III, a marker of fibrosis, demonstrated less staining in the cortical interstitium of margatoxin-treated CRF rat kidneys (Figure 3B(b) versus 3B(a)), indicating that margatoxin reduced the progression of renal fibrosis. The cortical expression of Cdk4 mRNA was significantly decreased after margatoxin treatment (Figure 3(C), left), and that of p21 mRNA was significantly increased after the treatment (right). These results suggested that the overexpression of Kv1.3 channels in leukocytes was strongly associated with the promotion of cell cycling, and thus with the progression of renal fibrosis.

## 4. Discussion

In the development of tubulointerstitial fibrosis in CRF rat kidneys, inflammatory leukocytes are initially recruited from the bone marrow and infiltrate into the renal interstitium to trigger the proliferation of fibroblasts [25]. Then, with the progression of uremia, the number of such leukocytes is considered to decrease due to the decrease in circulating lymphocyte counts [26]. In advanced CRF, however, the present study demonstrated that the numbers of leukocytes in the cortical interstitium were dramatically increased by *in situ* proliferation, showing pathological features similar to those of acute glomerulonephritis [27]. Since the cytokines produced by leukocytes stimulate the activity of fibroblasts to produce collagen [25], the increased number of leukocytes in the interstitium would promote the progression of renal fibrosis and thus contribute to the rapid deterioration of renal function in advanced CRF.

Previous studies have demonstrated the overexpression of Kv1.3-channnels in cells under various pathologic conditions, including cancer [28, 29] and ischemic heart disease [30]. Concerning the mechanisms involved in such overexpression of the channels, stimulation by transforming growth factor-*β* (TGF-*β*) was one of the most likely candidates demonstrated in macrophages [31]. Since uremic toxins, such as indoxyl sulfate, upregulate the expression of TGF-*β* [32], they might be responsible for the overexpression of the channels in leukocytes in advanced CRF. By generating a driving force for the calcium influx, Kv1.3 channels expressed in lymphocytes trigger the calcium signaling necessary for cellular proliferation [15, 16]. From our results, as demonstrated in cancer cells [33], the membrane hyperpolarization induced by the overexpression of the channels would also promote the proliferation of leukocytes by directly stimulating cell cycle progression.

In addition to their role in cellular proliferation, Kv1.3 channels expressed in lymphocytes and macrophages trigger the cytokine production from those cells [14–16]. Therefore, the overexpression of the channels in leukocytes would have multipliable effects on the progression of renal fibrosis. A previous study has demonstrated the therapeutic efficacy of blocking the intermediate-conductance Ca^2+^-activated K^+^-channels (Kc_a_3.1) for renal fibrosis, since fibroblasts overexpressed the channels under the pathologic condition [34]. From our results, targeting the Kv1.3 channels overexpressed in leukocytes would also be useful for the treatment of renal fibrosis in advanced CRF. In addition to the selective blockers for the channel that have previously been developed [35], we have recently demonstrated the inhibitory effects of nonsteroidal anti-inflammatory drugs (NSAIDs), such as diclofenac sodium, salicylate, and indomethacin, on Kv1.3 channels expressed in lymphocytes [36]. Concerning such properties, these drugs would potentially be useful as antifibrotic agents in patients with advanced CRF.

In summary, this study demonstrated for the first time that the number of leukocytes was dramatically increased in rat kidneys with advanced CRF. The overexpression of Kv1.3 channels in the leukocytes was thought to contribute to the progression of renal fibrosis by stimulating cell cycling and promoting cellular proliferation.

## Figures and Tables

**Table 1:** Primers used for quantitative real-time reverse transcriptase-PCR.

Gene	Primer	Product length (bp)
CD3	Forward: CAAAGAAACTAACATGGAGCAGGG	120
	Reverse: CTTTTTGCTGGGCCATGGT	
ED-1	Forward: TGTACCTGACCCAGGGTGGAA	92
	Reverse: GAATCCAAAGGTAAGCTGTCCGTAA	
KCNA3	Forward: GCTCTCCCGCCATTCTAAG	141
	Reverse: TCGTCTGCCTCAGCAAAGT	
Cdk4	Forward: CAATGTTGTACGGCTGATGG	120
	Reverse: GGAGGTGCTTTGTCCAGGTA	
p21	Forward: AGCAGTTGAGCCGCGATT	124
	Reverse: CGAACACGCTCCCAGACG	
GAPDH	Forward: GGCACAGTCAAGGCTGAGAATG	143
	Reverse: ATGGTGGTGAAGACGCCAGTA	
